# Evaluation of Operative Vaginal Delivery Practices and Maternal-Neonatal Outcomes

**DOI:** 10.7759/cureus.99392

**Published:** 2025-12-16

**Authors:** Falak Baloch, Maryam Javed, Liliana Grosu

**Affiliations:** 1 Department of Obstetrics and Gynaecology, Bedfordshire Hospitals NHS Foundation Trust, Bedford, GBR

**Keywords:** ctg, fetal distress, forceps, major obstetric hemorrhage, operative vaginal delivery, perineal tear, vacuum

## Abstract

Background

Operative vaginal deliveries (OVDs), including vacuum and forceps-assisted births, are essential interventions for prolonged second-stage labor or fetal compromise. Despite declining global rates, adherence to clinical guidelines and proper documentation remains crucial for maternal and neonatal safety. This audit aimed to evaluate operative vaginal delivery practices at Bedford Hospital, including adherence to Royal College of Obstetricians and Gynaecologists (RCOG) and local guideline recommendations and associated maternal and neonatal outcomes. The audit also aimed to identify areas for improvement to enhance patient safety and quality of care.

Methodology

A retrospective audit included all women (n = 62) undergoing attempted OVDs from September to December 2024. Data were extracted from electronic maternity records and NerveCenter, assessing instrument type, success rate, decision-to-delivery interval, documentation (consent, indication, debrief), consultant presence, antibiotic and venous thromboembolism (VTE) prophylaxis, maternal complications, and neonatal outcomes. Audit standards were based on RCOG Green-top Guideline No. 26 and local protocols.

Results

Monthly OVD rates ranged from 9/225 (4%) in September, 16/211 (7.58%) in October, 25/198 (12.6%) in November, to 12/224 (5.36%) in December, with a total of 62/858 (7.2%) OVDs and a 100% procedural success rate. Indications for instrumental delivery were documented in 60/62 (96.8%) cases, and consent was documented in 96.8% of cases, either written (for theatre deliveries) or verbal (for delivery-suite births). Vacuum and forceps were used equally (29/62, 46.8% each), with 4/62 (6.4%) sequential instrument cases. Registrars performed 57/62 (91.9%) deliveries, with consultants present in 22/62 (35.5%) cases. Maternal outcomes included 4/62 (6.45%) third-degree perineal tears and 8/62 (12.9%) cases of blood loss >1,000 mL, including 2/62 (3.2%) massive obstetric hemorrhages (1,940-2,200 mL). Neonatal outcomes were generally favorable, with 2/62 (3.2%) special care baby unit admissions, 4/62 (6.5%) shoulder dystocia events, and 2/62 (3.2%) low APGAR scores (<5 at birth or <7 at five minutes). Areas for improvement were identified in post-procedure debriefing (10/62, 16.1%), antibiotic prophylaxis (44/62, 70.9%), and postnatal VTE assessment (56/62, 90.3%).

Conclusions

OVDs in this cohort were performed safely with high success rates and appropriate indications. Rare but significant complications highlight the need for timely escalation, senior review, structured debriefing, and ongoing audit to optimize maternal and neonatal safety and maintain guideline adherence.

## Introduction

Operative vaginal delivery (OVD) refers to a vaginal delivery in which forceps or a vacuum device are used to assist the birth [1]. Indications for performing an OVD include a prolonged second stage of labor, evidence or risk of fetal distress, and the need to expedite delivery to assist the mother [2].

Instrumental deliveries account for 10-15% of all births and are associated with higher maternal and neonatal morbidity, which contributes to an increased risk of litigation [3]. Instrumental deliveries carry an increased risk of maternal and neonatal injury. Maternal complications may result in significant functional issues, such as fecal incontinence, which can have a profound impact on quality of life. Therefore, it is essential to obtain informed consent before any medical procedure [4].

Rates of OVDs have been declining globally, with a particularly marked reduction in the use of obstetric forceps. This trend has occurred alongside rising cesarean section rates worldwide, raising concerns about the diminishing proficiency in OVD techniques as cesarean delivery (CD) becomes increasingly favored [5]. Over the past three decades, CD rates and the incidence of associated accreta spectrum disorders have risen steadily, while the use of OVD and trials of labor after cesarean have declined. The effects of obstetric surgery on reproductive outcomes and future fertility options remain clinically significant [6].

According to the Royal College of Obstetricians and Gynaecologists (RCOG) Green-Top Guideline No. 26 (April 2020), it is crucial to know when to discontinue the procedure, the maximum number of attempts or pulls permitted, and the point at which the instrument should be abandoned to ensure patient safety [7]. The safe and effective use of OVDs requires adherence to established guidelines, proper training, and continuous audit of clinical outcomes.

Audit and research on OVD practices help identify gaps in clinical practice, assess compliance with guidelines, and improve maternal and neonatal outcomes. This study aimed to evaluate the patterns, indications, and outcomes of OVDs in our institution over a specified period, providing insights into current practice and areas for quality improvement. This audit was conducted at Bedford Hospital, part of the Bedfordshire Hospitals NHS Foundation Trust in the East of England. The audit standards were based on the RCOG Green-top Guideline No. 26 as well as local trust guidelines.

## Materials and methods

This was a retrospective audit conducted at Bedford Hospital, part of the Bedfordshire Hospitals NHS Foundation Trust in the East of England, over a four-month period from September to December 2024. All women who underwent attempted instrumental vaginal delivery during the study period were included, regardless of whether the procedure was successful. No specific exclusion criteria were applied. A total of 62 women met the inclusion criteria. A consecutive non-probability sampling approach was used, including all eligible cases during the audit period.

Data were extracted from electronic records (Mediviewer), including maternity and delivery notes, with additional information regarding antibiotic administration and venous thromboembolism (VTE) prophylaxis (Tinzaparin) obtained from the NerveCenter system. The audit assessed adherence to specific parameters outlined in the RCOG Green-top Guideline No. 26 (April 2020) and local trust protocols. These parameters included documentation of indication for OVD, type of instrument used, number of pulls, procedural success, decision-to-delivery intervals, operator grade, consultant presence, written consent, post-procedure debriefing, administration of antibiotic prophylaxis, and completion of postnatal VTE assessment. Maternal outcomes assessed included third-degree perineal tears, estimated blood loss >1,000 mL, and massive obstetric hemorrhage. Neonatal outcomes included special care baby unit (SCBU) admission, shoulder dystocia, APGAR scores, arterial pH, and birth weight.

Decision-to-delivery intervals were evaluated against standards: ≤30 minutes for cardiotocography concerns and ≤75 minutes for prolonged second stage of labor. Maternal and neonatal outcomes, documentation quality, and adherence to RCOG Green-top Guideline No. 26 and local trust guidelines were also assessed. Compliance with RCOG Green-top Guideline No. 26 and local trust guidelines was assessed for all parameters [7]. All parameters were pre-defined to ensure that the audit could be reproduced in other settings using the same standards.

Data were analyzed descriptively, with categorical variables presented as frequencies and percentages and continuous variables summarized using mean ± standard deviation where applicable. Ethical approval for the audit was granted by the Bedford Hospital audit department, and individual patient consent was not required due to the retrospective nature of the study and anonymization of data.

## Results

**Figure 1:**
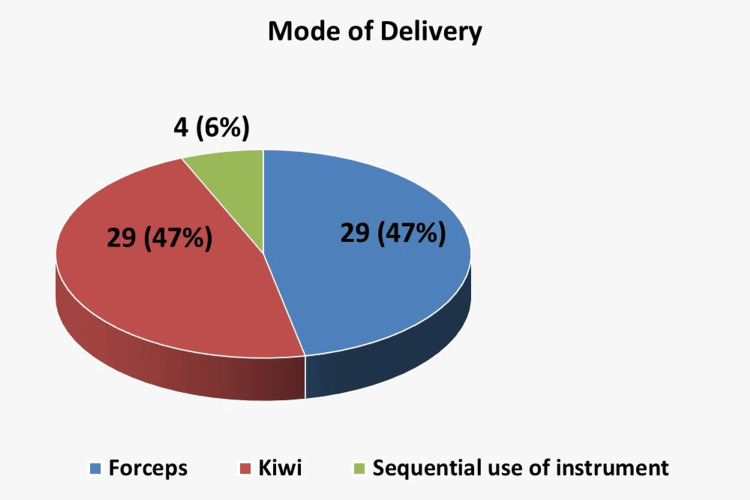
Distribution of operative vaginal deliveries by instrument type (vacuum, forceps, sequential use).

Between September and December 2024, 62 OVDs were performed. The monthly rate of instrumental deliveries was 9/225 (4%) in September, 16/211 (7.58%) in October, 25/198 (12.6%) in November, and 12/224 (5.36%) in December. All procedures were successful, achieving a 100% (62/62) success rate. The indication for instrumental delivery was documented in 96.77% (60/62) of cases, with only two instances not documented by the doctor but noted in the midwife’s records. The distribution of indications is illustrated in Figure 1, showing the proportion of cases due to delay in the second stage of labor, suspected fetal hypoxia, or both.

Overall, decision-to-delivery intervals ranged from 3 to 73 minutes. Five (5/62, 8.1%) cases did not meet the recommended decision-to-delivery interval criteria. Of these, one case was delayed due to temperature issues in the theater; an alternative theater was arranged, the incident was reported, and later investigated. Another case was delayed because an instrument was initially applied but removed due to absent contractions; oxytocin was subsequently initiated, and the instrument was reapplied. For the remaining three cases, no specific cause for the delay could be identified from the documentation.

**Figure 2:**
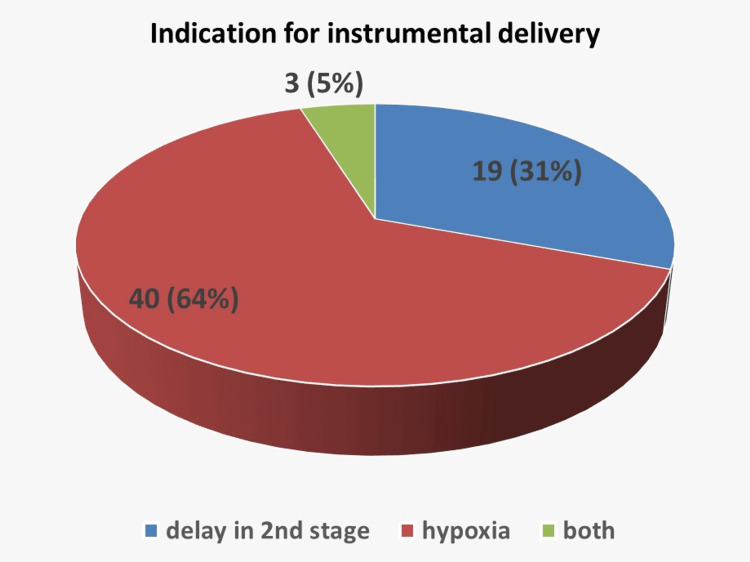
Pie chart showing the distribution of indications for operative vaginal delivery.

Vacuum and forceps were used equally, with 29 cases each (29/62;46.8%), while sequential instruments were required in four (4/62; 6.4%) cases, as shown in Figure 2. Registrars performed 57 of the deliveries (57/62; 91.9%), and consultants performed five (5/62; 8.1%); a consultant was present in 22 (22/62; 35.5%) cases. Overall, 39 deliveries occurred in the delivery suite room (39/62; 62.9%), while 23 were conducted in theatre (23/62; 37.1%). All doctors performing the procedures were deemed competent. Appropriate trial of instrumental delivery was ensured in cases with occiput posterior, occiput transverse, or high head with caput/moulding. Indications for instrumental delivery were documented in 60/62 (96.8%) cases, and consent was documented in 96.8% of cases, either written (for theatre deliveries) or verbal (for delivery-suite births). The operative details, maternal outcomes, and neonatal outcomes are summarized in Table 1.

**Table 1:** Operative details and maternal and neonatal outcomes of women undergoing operative vaginal delivery (n = 62). The table summarizes operative details, maternal outcomes, and neonatal outcomes for the 62 women who underwent operative vaginal delivery. Categorical variables are expressed as n (%), and continuous variables are expressed as mean ± SD where applicable. SCBU: special care baby unit; MOH: massive obstetric hemorrhage; VTE: venous thromboembolism

Characteristic	Category/Measurement	n (%) or mean ± SD
Mode of instrument	Vacuum	29 (46.8%)
	Forceps	29 (46.8%)
	Sequential	4 (6.4%)
Operator	Registrar	57 (91.9%)
	Consultant	5 (8.1%)
Consultant presence	Present	22 (35.5%)
	Not present	40 (64.5%)
Delivery location	Delivery suite	39 (62.9%)
	Theater	23 (37.1%)
Maternal outcomes	Third-degree perineal tear	4 (6.45%)
	Estimated blood loss >1,000 mL	8 (12.9%)
	MOH	2 (3.2%)
Antibiotic prophylaxis	Given	44 (70.9%)
	Not given	18 (29.1%)
Postnatal VTE assessment completed	Yes	56 (90.3%)
	No	6 (9.7%)
Debrief documented	Yes	10 (16.1%)
	No	52 (83.9%)
Neonatal outcomes	SCBU admission	2 (3.2%)
	Shoulder dystocia	4 (6.5%)
	APGAR <5 at birth	1 (1.6%)
	APGAR <7 at 5 minutes	1 (1.6%)
	Arterial pH <7.10	3 (4.8%)
	Birth weight >4 kg	8 (12.9%)
Maximum pulls required	1–4 pulls	1 case with 4 pulls (Kiwi vacuum)

Maternal outcomes included four third-degree perineal tears (6.45%) and estimated blood loss over 1,000 mL in eight women. Two women experienced massive obstetric hemorrhage, which is described below to highlight learning points.

Case 1, with massive obstetric hemorrhage of 2,200 mL, was a 31-year-old primiparous, white British woman with a body mass index (BMI) of 25.9 kg/m² who underwent a Kiwi vacuum delivery for a full-term pregnancy at 09:05 with two pulls. The baby was born in good condition. The placenta was delivered at 09:44, but the cord snapped during controlled cord traction, and the placenta appeared incomplete. Two additional pieces of placenta were removed on vaginal examination. Despite administration of oxytocin, ergometrine, tranexamic acid, and a single dose of hemabate, bleeding continued. A code red was called at 1,000 mL estimated blood loss due to uterine atony and retained placenta. Bimanual compression was performed. Pre-delivery hemoglobin was 118 g/L, and post-delivery hemoglobin was 63 g/L; one unit of red blood cells was transfused. Surgery for manual removal of the placenta was delayed due to another ongoing emergency and consent process. Surgery was performed in the theater under spinal anesthesia at 11:32. She recovered well, but no debrief or senior review was documented. Learning point from this case was to escalate promptly to the consultant and matron on call.

Case 2, with massive obstetric hemorrhage of 1,940 mL, was a 30-year-old primiparous woman with a BMI of kg/m² who underwent a forceps delivery with episiotomy at 17:51 due to fetal tachycardia. Two pulls were performed in the delivery room, and the baby was born in good condition. Postpartum hemorrhage was both traumatic and atonic. Management included oxytocin bolus, ergometrine, tranexamic acid, and one dose of hemabate. Episiotomy suturing was completed at 18:24, and bleeding was brought under control. Learning point from this case was to improve monitoring of estimated blood loss, escalate promptly, initiate a massive obstetric hemorrhage call, and document debrief.

Sequential instruments were successfully used in four women, with only one neonate requiring SCBU admission. Antibiotic prophylaxis was administered in 70.9% of women, while postnatal VTE assessments were completed in 90%. Debriefing was documented in only 16% of cases.

Neonatal outcomes were favorable overall. Two babies were admitted to the SCBU, four experienced shoulder dystocia, and one had an APGAR score <5 at birth, while another had an APGAR score <7 at five minutes. Three babies had an arterial pH <7.10, and eight had a birth weight above 4 kg. The maximum pulls recorded were four, with only one case requiring four pulls using a Kiwi vacuum.

Patient demographics showed 47 primiparous women (47/62; 75.8%), 11 para 1 (11/62; 17.7%), 3 para 2 (3/62; 4.8%), and 1 para 3 (1/62; 1.6%). The majority (45) had gestational ages between 37 and 41 weeks, nine between 34 and 37 weeks, and eight between 41 and 42 weeks. BMI distribution included three women <20 kg/m², 25 women 20-25 kg/m², 26 women 26-30 kg/m², and 11 women >30 kg/m². Induction of labor was performed in 26 (26/62; 41.9%) women, with the remaining 36 undergoing spontaneous onset of labor (36/62; 58.1%). Analgesia was appropriately provided in all cases, with 29 women receiving Entonox (29/62; 46.8%) and 10 receiving pethidine (10/62; 16.1%). Patient demographics and clinical characteristics are summarized in Table 2.

**Table 2:** Clinical and demographic characteristics of women undergoing operative vaginal delivery (n = 62). This table summarizes the baseline characteristics of the study cohort, including parity, gestational age, maternal BMI, onset of labor, analgesia, mode of instrumentation, consultant presence, and delivery location. Percentages are calculated for categorical variables, and mean ± SD is reported where applicable. BMI: body mass index

Characteristic	Category/Measurement	n (%) or mean ± SD
Parity	Primiparous	47 (75.8%)
	Para 1	11 (17.7%)
	Para 2	3 (4.8%)
	Para 3	1 (1.6%)
Gestational age (weeks)	34–37	9 (14.5%)
	37–41	45 (72.6%)
	41–42	8 (12.9%)
Maternal BMI (kg/m²)	<20	3 (4.8%)
	20–25	25 (40.3%)
	26–30	26 (41.9%)
	>30	11 (17.7%)
Labor onset	Spontaneous	36 (58.1%)
	Induced	26 (41.9%)
Analgesia	Entonox	29 (46.8%)
	Pethidine	10 (16.1%)
Mode of instrument	Vacuum	29 (46.8%)
	Forceps	29 (46.8%)
	Sequential	4 (6.4%)
Consultant presence	Present	22 (35.5%)
	Not present	40 (64.5%)
Delivery location	Delivery suite	39 (62.9%)
	Theater	23 (37.1%)
Maternal outcomes	Third-degree perineal tear	4 (6.45%)
	EBL >1,000 mL	8 (12.9%)
Neonatal outcomes	SCBU admission	2 (3.2%)
	Shoulder dystocia	4 (6.5%)
	APGAR <5 at birth	1 (1.6%)
	APGAR <7 at 5 minutes	1 (1.6%)
	Arterial pH <7.10	3 (4.8%)
	Birth weight >4 kg	8 (12.9%)

Overall, the audit demonstrated that OVDs in this cohort were conducted safely with high success rates and appropriate indications. Areas for improvement included documentation of debriefing, postnatal VTE assessment completion, and ensuring antibiotic prophylaxis adherence. The two massive obstetric hemorrhage cases emphasize the importance of timely escalation, senior review, and structured debriefing to optimize patient safety.

## Discussion

This audit reviewed 62 OVDs over a four-month period. The overall rate of instrumental deliveries ranged from 4% to 12.6% monthly, which aligns with national and international reports indicating rates ranging between 5% and 15% depending on the obstetric population and institutional practice [7,8]. The 100% procedural success rate demonstrates safe and effective practice, consistent with studies showing that with proper training, OVDs can be performed with high success and low morbidity [9]. This aligns with recent studies indicating high success rates for vacuum-assisted deliveries, with one study reporting a 97.3% success rate for vacuum attempts [10].

Documentation of indications and consent was achieved in most cases (96.77% and 92%, respectively), although this remains similar to the previous audit, indicating persistent gaps in record-keeping. A recent study highlighted that documentation compliance remains a challenge, with many OVDs lacking complete documentation, including indications and consent [11]. This underscores the need for standardized documentation practices to enhance clinical accountability and communication.

Compliance with decision-to-delivery intervals was comparable to the previous audit (92% versus 93%), which is in line with current guidelines recommending timely intervention in second-stage complications [12]. Maternal outcomes were largely favorable, with low rates of third-degree perineal tears (6.45%), and most patients experienced manageable blood loss. While two cases of massive obstetric hemorrhage were observed, it is important to note that these are recognized complications that can also occur in spontaneous vaginal deliveries. Therefore, these events cannot be attributed solely to the use of instruments but highlight the importance of prompt escalation, senior review, and structured debriefing, as recommended in national guidelines for the management of postpartum hemorrhage [13]. Recent guidelines emphasize the use of a treatment bundle for the management of postpartum hemorrhage, which was followed in these cases, including uterotonics, tranexamic acid, and escalation of care if bleeding persists [14].

Sequential instruments were used safely in four women, all resulting in successful deliveries, demonstrating that complex operative techniques can be performed effectively with appropriate supervision [15]. This is consistent with recent studies indicating that when performed by trained professionals, sequential instrument deliveries can be safe and effective [12].

Neonatal outcomes were reassuring, with minimal need for SCBU admission and isolated cases of low APGAR scores or shoulder dystocia. These findings are consistent with previous studies indicating that OVD, when appropriately indicated and performed by trained staff, is generally safe for the neonate [16,17]. Areas for improvement identified include post-procedure debriefing (documented in only 16%), completion of postnatal VTE assessment (90%), antibiotic prophylaxis adherence (70.9%), and paired cord gas collection (77.4%). Addressing these gaps aligns with best-practice recommendations and will further enhance patient safety and quality of care.

Recommendations

Based on the findings of this audit, several measures are recommended to improve the safety and quality of OVDs. Implement the use of a delivery categorization sticker to classify deliveries, operative vaginal or cesarean, based on urgency (Category 1 or 2) and indicate the recommended location (delivery suite room or theatre). This tool will support timely decision-making, ensure appropriate escalation of care, and improve adherence to recommended decision-to-delivery intervals in line with RCOG guidelines. Although the sticker is copyright-protected and cannot be shared externally, its implementation is intended to further enhance compliance with guidelines and support quality improvement in future audits. The delivery categorization sticker has been implemented to improve decision-making and adherence to decision-to-delivery intervals.

A standardized documentation template should be developed to capture essential details, including in-room consent, a clear indication for the procedure, and a structured post-procedure debrief, which will help address persistent gaps in record-keeping. Antibiotic prophylaxis should be administered consistently as a single dose post-delivery when indicated, and paired cord gases should be collected for all babies to ensure comprehensive neonatal assessment and documentation. Re-audit in six months is recommended to evaluate the effectiveness of these interventions and monitor ongoing compliance with best practice standards.

Limitations

This audit has a few limitations. As it was conducted at a single center, the findings may not apply to other hospitals. Only short-term maternal and neonatal outcomes were assessed, and no long-term follow-up was performed. It is important to note that the primary aim of this audit was to evaluate adherence to guidelines and recommendations for OVDs, including documentation, decision-to-delivery intervals, and immediate procedural outcomes. Long-term follow-up of women who sustained obstetric anal sphincter injuries was not within the scope of this audit, but can be explored in a separate prospective study focusing on maternal recovery and patient perceptions after OVD.

## Conclusions

This audit demonstrated that OVDs in this cohort were performed safely, with high success rates and appropriate indications. Maternal and neonatal outcomes were generally favorable, including in cases requiring sequential instruments. However, rare but significant complications, such as massive obstetric hemorrhage, underscore the importance of timely escalation, senior review, and structured debriefing. Focused efforts on improving documentation, adherence to post-delivery protocols, and systematic monitoring will help maintain and further enhance the quality of care.
